# Presentation, aetiology and treatment of adult intussusception in a tertiary Sub-Saharan Hospital: a 10-year retrospective study

**DOI:** 10.1186/1471-230X-14-86

**Published:** 2014-05-05

**Authors:** Peter A Ongom, Christopher K Opio, Stephen C Kijjambu

**Affiliations:** 1 Colorectal Unit, Department of Surgery, School of Medicine, Makerere College of Health Sciences, Makerere University, PO Box 7072, Kampala, Uganda; 2 Gastroenterology Unit, Department of Internal Medicine, School of Medicine, Makerere College of Health Sciences, Makerere University, PO Box 7072, Kampala, Uganda; 3 Department of Surgery, School of Medicine, Makerere College of Health Sciences, Makerere University, PO Box 7072, Kampala, Uganda

**Keywords:** Adult intussusception, Sub-acute and chronic symptoms, Tumour, Malignancies, Lead point, Idiopathic, Reduction, Resection

## Abstract

**Background:**

Adult intussusception is a rare clinical condition worldwide. It contributes to less than 5% of all cases of intussusception. Few studies have been conducted in low-income countries compared to high-income countries; particularly Sub-Saharan Africa. Based on anecdotal evidence, the authors hypothesized that the condition is not as rare in a Sub-Saharan setting in comparison with western countries. We set out to conduct the first review study of adult intussusception in Uganda.

**Methods:**

The medical records of 37 (out of a total of 62 cases) adolescent and adult patients with a postoperative diagnosis of intussusception at Mulago National Referral and Teaching Hospital, from January 2003 to December 2012, were analyzed. The clinical features, diagnosis, treatment and pathologic features of lesions for these patients were reviewed. Intraoperative findings were described with reference to: the site of the intussusception, and the triggering lesion (either idiopathic or with a lead point).

**Results:**

The mean age was 33.6 years, with a range of 13 – 72 years. The male to female ratio was 1.85:1. The mean number of days for which symptoms had been present prior to presentation was 6.3 days, while the median was 4 days. All 37 patients presented with abdominal pain. Only 13 (35.1%) had the classical paediatric triad of abdominal pain, a palpable abdominal mass and bloody stool. Most of the remaining patients presented sub-acutely with non-specific symptoms. A lead point was present in 28 patients (75.7%). Of these, 24 (64.9%) cases involved tumours. Among the tumours, 54.2% were malignant. Treatment did not involve intussusception reduction in 14 patients (37.8%). Some form of operative surgery was conducted in 31 (83.8%) patients; mainly segmental bowel resections and hemi-colectomies.

**Conclusion:**

Adult intussusception is uncommon in the Uganda, though probably less so than in western countries. It presents sub-acutely or chronically and is often diagnosed at laparotomy. Lead points are the triggering lesion most times and are due mainly to tumours. The bulk of tumours are malignant. Most patients require surgical resection, with prior reduction done in selected cases.

## Background

Intussusception is the invagination of a segment of the intestine into the lumen of another immediately adjacent segment. This is usually in a proximal-to-distal fashion. Adult intussusception (AI) is relatively uncommon, constituting less than 5 percent of intussusception cases [1]. In high-income countries, there is an incidence of two to three per 1,000,000 per year representing 1 to 3 percent of all cases of intestinal obstructions [2,3]. It is estimated that only 5% of all intussusceptions occur in adults and approximately 5% of bowel obstructions in adults are the result of intussusception [4]. There is a demonstrable cause in the majority of cases, usually an intraluminal neoplasm. Previous studies point to a 70 to 90 percent existence of an underlying gut pathological cause [2,3,5]. These are mainly polyps and colonic malignancies. In contrast, childhood intussusception is a leading cause of intestinal obstruction.

The pathophysiologic mechanism is the peristaltic movement of a lesion associated with the intussusceptum (invagination). Intussusceptions with no clear anatomical lesion are the primary or idiopathic type, present in 8 to 20% of cases, and more likely to occur in the small intestines [2,6,7]. In contrast, the secondary type, constituting the greater majority, is due to an existing gut pathological lesion. This lesion is best described as a lead point; a functional or structural lesion associated with the intussusceptum, postulated to be the “trigger” for intussusception. The commonest “lead points” are colonic malignant tumors, present in up to 60 percent of cases [2,4,5,8,9]. Benign tumours constitute the majority of the rest.

The epidemiology of this condition in low-income, Sub-Saharan African countries has also been documented. In one Nigerian study, intussusception was responsible for 8% of intestinal obstruction cases [10]. A study in a Kenyan centre described ileocolic intussusception as the 4^th^ commonest cause of bowel obstruction [11]. Another Nigerian study described a male to female ratio of 1.4:1, with a mean age of 49.6 years [12]. Seventy seven percent of cases had definite causes identified; mainly polyps (31.8%) and colonic malignancies (18%). Ileocolic intussusception was the commonest variety. Bowel resection for colonic carcinoma, gangrenous bowel and irreducibility of the intussusception was done for 72.7% of patients, while manual reduction was successful in 27.3%.

A 3-year Ethiopian study identified 2 peak ages of occurrence; the 2^nd^ and 4^th^ decades, associated mainly with idiopathic and secondary intussusception respectively [13]. The ileocolic type was present in 56% of cases. Benign conditions represented the majority (67%) of the identified lead points. Intraoperative reduction was successful in only 24% of the cases, all of which were idiopathic.

AI often presents with non-specific symptoms. The majority of cases have been reported as chronic, a symptom consistent with partial obstruction [2,14]. The classic triad of crampy abdominal pain, bloody (“currant jelly”) stool, and a palpable mass of acute intussusception (characteristic of paediatric presentation), is rare. The predominant symptoms are those associated with some form of bowel obstruction, and are most times described as non-specific. These include: abdominal pain and distension, nausea, vomiting, gastrointestinal bleeding, constipation, and changes in bowel habits [8,15].

Preoperative diagnosis remains difficult, while whether the intussusception should be reduced, and the extent of resection, remains controversial [4]. The optimal surgical approach in adult intussusception is also debatable. More recently, manual reduction of the intussusception followed by definitive surgical resection has been advocated. Studies dating back 5 decades ago recommended primary resection without attempting reduction in all adult patients with intussusception, regardless of anatomic site, because of significant risk of associated malignancy [16]. This point was echoed more recently in a series in which malignancy was the cause in 65% of intussusceptions [8]. Thus, a controversy continues to focus on whether AI should be surgically resected without an attempt at reduction, for fear that undue operative manipulation of a malignant lesion may result in tumour dissemination [17]. Sub-Saharan Africa authors also describe the need for laparotomy for virtually all cases [12], with subsequent reduction. En-block resection of the involved intestinal segment is recommended if there is obvious ischemia, while limited resection is recommended if a only a lead point is identified [13].

We describe our experience of adult intussusception, and discuss the clinical presentation, aetiology and optimal surgical management in a large, tertiary hospital in Sub-Saharan Africa.

## Methods

This was a retrospective study covering a 10-year period. The medical records of 62 adolescent and adult patients (13 years and above) with a postoperative diagnosis of intussusception at Mulago National Referral and Teaching Hospital, from January 2003 to December 2012, were collected. This hospital is located in Kampala, Uganda’s capital city. Permission to carry out the study was granted by Mulago Hospital’s Research and Ethics Committee. The clinical features, diagnosis, management and pathology of the 62 patients were reviewed. Twenty five patients were excluded from the study on account of incomplete or unclear records. Important information which led to these exclusions was that pertaining to: age, clinical features, intraoperative findings and procedure, and histological findings (when indicated). Operative procedures which did not involve resection did not necessarily have to have histology results for inclusion. Overall, we analyzed the records of 37 patients. The intraoperative findings were described in two contexts. One was the site of the intussusception, the other was the triggering lesion. The triggering lesion was descibed as either idiopathic or a structural, pathological lead point.

An intussusception that involved only the jejunum or ileum was considered an enteric intussusception. That which involved the ileum and the colon was designated as an ileocolic intussusception, while that involving only the colon was considered a colocolonic intussusception [4]. More detailed site description was made to suit unique individual presentations, for example, sigmoidorectal intussusception. Procedures carried out were described according to the surgeon’s notes. Essentially there was bowel reduction, with or without resection, for all cases. Resection was either segmental or a colectomy. Patients records where followed-up for up to 2 weeks postoperative or up to the time of discharge, whichever came first. Available records were entered into Microsoft™ spread sheets and exported to Stata® version 10 (StataCorp, Texas, USA) for detailed analysis. We generated descriptive stastistics that included frequencies/proportions, and measures of dispersion and central tendency.

## Results

A total of 37 patient’s records were analyzed. All diagnoses of intussusception were made intra-operatively. Of these, 24 (64.9%) were males and 13 (35.1%) females. The male to female ratio was 1.85:1 (Table 1). The mean age was 33.6 years, with a range of 59 (13 – 72) years. The 50^th^ percentile was 33 years and the interquartile range was 13 years, which meant that most patients were between the age of 20 and 46 years. The dominant ethnic group involved was the Ganda, 18 patients (48.65%); other ethnic groups, 11 in number, each contributed between 1 and 3 patients. Most patients (21; 56.8%) resided in Kampala (Uganda’s Capital city) and Wakiso districts.

**Table 1:** Summaries demographics of participants

**Patient demographics**	
Total patients	37
Mean age (years)	33.6
Age range (years)	13 – 72
50th percentile; age (years)	33
Interquatile range; age (years)	13
Male: female ratio	1.85:1
Mean duration of symptoms (Days)	6.3
Median duration of symptoms (Days)	4
Benign: malignant	11:13
Demonstrable lead point: idiopathic	3.1:1

All patients were symptomatic, with symptoms lasting from between 1 to 35 days. The mean duration for which symptoms lasted prior to presentation was 6.3 days (SD 7.59), with a 50^th^ percentile of 4 days. All 37 patients had a history of pain. The next two most frequent symptoms were nausea and vomiting, present in 35 (94.6%) and 28 (75.7%) patients respectively (Table 2). An abdominal mass was present in 22 (59.5%) patients. One patient had an ileocolic intussusception with anal protrusion [18], while another had a sigmoidorectal intussusception in combination with a rectal prolapse. Overall, the patients had an averagely sub-acute presentation, going by a popular classification [19].

**Table 2:** Distribution of clinical features

**Clinical feature**	**Number (%)**	**CI (%)**
Pain	37 (100)	88.8 – 100
Nausea	35 (94.6)	81.4 – 99.4
Vomiting	28 (75.7)	59.7 – 86.8
Constipation	18 (48.7)	33.5 – 64.1
Distension	24 (64.9)	48.7 – 78.2
Haematochezia	21 (56.8)	40.9 – 71.3
Diarrhoea	8 (21.6)	11.1 – 37.4
Abdominal mass	22 (59.5)	43.5 – 73.7
Anal protrusion	2 (5.4)	0.6 – 18.6

Shows the frequencies of symptoms and signs manifested by patients in the study.

Both the ileum and the colon (ileocolic area) were involved in 16 (43.2%) patients, making that the commonest site (Table 3). Seven (18.9%) patients had only the small intestines affected. More specifically, the prominent sites involved in descending order of frequency were: ileocaecal, ileocolic, ascending colon, and transverse colon, with 8, 6, 4, and 3 patients respectively. A total of 28 cases (75.7%) had a demonstrable lead point. Majority of intussusceptions were initiated by adenocarcinoma and idiopathic causes, each having 9 patients, and totaling 18 (48.6%) patients (Table 4). All patients with adenocarcinoma had the lesion either in the colon or the ileocolic region; none being purely enteric. Overall, tumours were the cause of intussusception in 24 patients (64.9%). Of all tumours, 13 (54.2%) were malignant, 3 of them being in the small intestines. Gangrene or necrosis was present in 10 (27.0%) patients.

**Table 3:** Summary of operative findings at laparotomy and surgical procedures done

**Index number**	**Histopathology/clinical diagnosis**	**Location**	**Necrosis/gangrene**	**Bowel reduction**	**Surgical procedure**
1	Idiopathic	Ileocaecal	Y	N	Segmental resection
2	Adenocarcinoma	Ileocaecal	N	C	Right hemicolectomy
3	Adenoma	Colocolic (ascending)	N	N	Right hemicolectomy
4	Non-specific inflammation	Ileoileal	Y	N	Segmental resection
5	Idiopathic	Ileocaecal	N	C	No resection
6	Idiopathic	Ileoileal	Y	C	Segmental resection
7	Adenocarcinoma	Colocolic (descending)	Y	N	Left hemicolectomy
8	Adenoma	Colocolic (ascending)	N	P	Right hemicolectomy
9	Idiopathic	Ileocaecal	Y	N	Right hemicolectomy
10	Adenocarcinoma	Ileocolic	N	C	No resection
11	Idiopathic	Ileocolic	N	C	No resection
12	Lymphoma	Ileoileal	Y	N	Segmental resection
13	Lipoma	Ileocaecal	N	P	Right hemicolectomy
14	Lymphoma	Ileoileal	N	C	Segmental resection
15	Lymphoma	Colocolic (transverse – sigmoid colon)	N	C	Left hemicolectomy
16	Kaposi’s sarcoma	Ileoileal	N	N	Segmental resection
17	Lipoma	Ileocaecal	N	C	Right hemicolectomy
18	Adenocarcinoma	Ileocaecal	N	C	Right hemicolectomy
19	Adenoma	Ileocaecal	N	C	Right hemicolectomy
20	Idiopathic	Ileocolorectal, anal protrusion [18]	N	P	Right hemicolectomy
21	Lipoma	Colocolonic (transverse), 'giant' lipoma; polyp [25]	N	P	Left hemicolectomy
22	Adenocarcinoma	Colocolic (ascending)	N	N	Right hemicolectomy
23	Adenocarcinoma	Colocolic (ascending)	N	N	Right hemicolectomy
24	Idiopathic	Ileocolic	Y	N	Segmental resection
25	Adenocarcinoma	Colocolic (transverse)	N	P	Extended right hemicolectomy
26	Adhesions	Ileocolic	Y	N	Segmental resection, Adhesiolysis
27	Adhesions	Ileoileal	N	C	Segmental resection, Adhesiolysis
28	Adenoma	Colocolic (ascending)	N	C	Right hemicolectomy
29	Adenocarcinoma	Colocolic (descending)	N	P	Sigmoid colectomy, hartmann's procedure
30	Adhesions	Ileoileal	N	C	No resection, adhesiolysis
31	Idiopathic	Ileocolic	Y	N	Segmental resection
32	Lipoma	Ileocolic	Y	N	Right hemicolectomy
33	Adenoma	Colocolic (transverse)	N	C	No resection, polypectomy
34	Idiopathic	Ileocolic, ileal - transverse colonic	N	C	Segmental resection
35	Adenocarcinoma	Colocolic (transverse)	N	N	Left hemicolectomy
36	Leiomyoma	Colocolic (transverse), polyp	N	C	Segmental resection
37	Adenoma	Colorectal, sigmoidorectal with rectal proplapse	N	C	Reduction and polyp biopsy

• Necrosis/Gangrene: Y – present, N – absent.

• Reduction: C – complete reduction done, P – partially reduced, N – no reduction attempted.

• [18] and [25] indicate published references.

**Table 4:** Lesions associated with intussusception

**Lesion/lead point**	**Number (%)**	**CI (%)**
**Benign causes**	13 (35.1)	21.8 – 51.3
Adhesions	3 (8.1)	2.1 – 22.0
Idiopathic	9 (24.3)	13.2 – 40.3
Non-specific inflammation	1 (2.7)	< 0.01 – 15.1
**Benign tumours**	11 (29.7)	17.4 – 45.9
Adenoma	6 (16.2)	7.3 – 31.5
Lipoma	4 (10.8)	3.7 – 25.3
Leiomyoma	1 (2.7)	< 0.01 – 15.1
Malignant tumours	13 (35.1)	21.8 – 51.3
Adenocarcinoma	9 (24.3)	13.2 – 40.3
Lymphoma	3 (8.1)	2.1 – 22.0
Karposi's sarcoma	1 (2.7)	< 0.01 – 15.1
Total	37 (100)	

In the treatment of the AI, complete bowel reduction was achieved in 17 (46.0%) patients (Table 3); surgical resection was not done. Five of these patients had only the small intestines involved. Six (16.2%) had partial reduction prior to resection, while in 14 patients (37.8%; CI 24.0 – 53.9) reduction was not attempted at all. Thirty one patients (83.8%; CI 68.5 – 92.7) underwent some form of surgical resection; 13 – right hemicolectomy, 12 (32.4%; CI 19.6 – 48.7) – segmental bowel resection, 4 – left hemicolectomy, and 1 – extended right hemicolectomy. One patient had a sigmoid colectomy in combination with Hartmann’s procedure (Table 3). Two patients presented with anal protrusion; one acutely, the other chronically. The patient with an acute presentation had precipitous symptoms lasting only minutes, before progressing to an acute intestinal obstruction. He had a sigmoidorectal type, with an adenoma as the lead point. Alongside this picture was a rectal prolapse. The prolapse/intussusception was reduced, and an incisional biopsy done. Histopathologic examination revealed it to be an adenoma. The patient with a chronic presentation had an ileocolic type, with an idiopathic cause. There was gross thickening and fibrosis, not allowing complete reduction. Resection had to be done. There was neither tumour nor gangrene. One patient with a transverse colonic polyp had a colonic incision made at the site of the pedunculated polyp, followed by polypectomy and minimal colonic resection, proximal and distal to the polyp’s stalk attachment. Histopathologic examination revealed a leiomyoma. One patient who had had a right hemicolectomy, developed an enterocutaneos fistula one week postoperative. He recovered on non-operative management. There were no deaths recorded.

## Discussion

We show that AI is not rare in this study setting; ages 13 to 72 years. A related Ethiopian study described the condition as not being rare in adults [13]. All our patients presented with pain. Other prominent symptoms were nausea and vomiting. Tumours formed 2/3 of the causes of AI. The study resulted in the analysis of 37 participants, with a male to female ratio of 1.85:1, a mean age of 33.6 years and a range of 59 (13 – 72) years. The mean number of days for which symptoms had been present prior to presentation was 6.3 days, while the median was 4 days. Only 13 (35.1%) patients had the classical paediatric triad of abdominal pain, a palpable abdominal mass and bloody stool. On average, most of the remaining patients presented sub-acutely with non-specific symptoms. A pathological lead point was present in 28 patients (75.7%). Of these, 24 (64.9%) cases involved tumours. Among the tumours, 54.2% were malignant. Treatment did not involve bowel reduction in 14 patients (37.8%). Surgical resection was conducted in 31 (83.8%) patients.

Generally AI is unique when compared to its paediatric form in that it is rare, accounting for only 5% of all cases of intestinal obstructions. The mechanism, though still unclear, is believed to be the result of any lesion in the bowel wall, or irritant within the lumen, that alters normal peristaltic activity, thereby initiating invagination.

As seen in other studies, our study had males more affected than females (1.85:1) [2,12]. However, some authors have reported reciprocal findings [17]. The mean age of 33.6 years is lower than that reported in some previous studies [2,12,20]. A related Sub-Saharan study reported 2 peak age groups; during the second and fourth decades [13]. Our mean age, within the fourth decade, is comparable to that study’s findings.

Ethnicity and residence are important to describe, since they illustrate the study setting we are in. The dominant ethnic group involved was the Ganda (Bantu), 18 patients (48.65%). This is plausibly explained by their being the dominant population in the greater Kampala region, in comparison to the multiple ethnic groups from the rest of the country [21]. Moreover, they are also the most populous ethnic group in the entire country. In turn, the majority of patients (21; 56.8%) resided in Kampala and Wakiso districts, both urban and semi-urban areas. These districts are the key catchment area for Mulago Hospital. We cannot comment on the association between ethnicity and AI from this study.

The clinical presentation of intussusception in this study varied considerably, as is the case in most other studies. Presenting symptoms were largely non-specific, and functionally leaned towards features of partial obstruction [2,14]. The mean duration for which symptoms lasted prior to presentation, 6.32 days, makes our series a sub-acute type [19]; 4 to 14 days duration. Symptom durations of < 4 days describe acute presentation, while durations > 14 days relate to chronicity. Seventeen (45.9%) patients, a sizeable proportion, presented with acute symptoms. This is in contrast to the mean duration for other studies which point to more chronic presentations; Azar et al. had a mean of 37.5 days [2], while Rathore et al. had a series with the shortest symptom duration being 2 weeks [20]. A possible explanation for this is the inclusion of adolescents (in our study) who have a shorter duration of symptoms, more related to the acute paediatric presentation, thus lowering the mean age for our entire group. The most common symptoms of pain, nausea and vomiting, were also observed in several other studies [1,2,4,5,20]. Haematochezia, diarrhoea, changes in bowel habits, constipation, and abdominal distension, are other non-specific symptoms and signs present in our series, yet also prominent in others [8,15]. The respective prevalences for all these are comparable (Table 1). Anal protrusion, which represented 5.4% of our cases, is a rare clinical feature, usually reported as individual case reports.

Previous studies have described abdominal masses as being palpable in 24% - 42% of patients [2,6,22]. The presence of a shifting mass or one that is palpable only when other symptoms are present is suggestive of intussusception. In our study this specific characteristic was not particularly noted. An abdominal mass was present in 59.5% (CI 43.5 - 73.7) of patients. We postulate that the inclusion of adolescents, who may have more of the classic presentation, with its characteristic “mass”, account for this higher percentage. Other studies have had a lower age limit of 18 years for participants. Furthermore, we still had 13 (35.1%; CI 21.8 – 51.3) patients with the classical paediatric traid, a higher proportion compared to another documented observation of 9.8% [9]. The adolescence factor could yet again account for this. The low proportion of classical presentation is one of the reasons why pre-operative diagnosis is difficult in these patients.

AI is also classified according to the presence or absence of a demonstrable lead point, and by the location of the lesion; enteric, colonic or border-line (ileocolic). Intussusception with an organic lesion as the lead point usually presents as a bowel obstruction, persistent or relapsing, necessitating definite surgical therapy. Our study had 24.3% (CI 13.2 - 40.3) of cases with idiopathic presentation, meaning no demonstrable lesion (Table 2). Though this is a higher prevalence, our study compares well with other study observations of 8 – 20% [2,6,7]. A demonstrable pathological lead point, the “hallmark” of AI, was seen in the majority (28 patients), constituting 75.7% (CI 60 – 86.8). Most documented studies also have their cardinal observation as the vast majority of cases being non-idiopathic, with percentages of 83.3% [19], 86.4% [4], 90% [5], 90.9% [9] and 93% [2] having been reported. Our study is comparable to these.

In our study, the ileocolic area was the most involved site, followed by the small intestines alone; a combined contribution of 62.2% of all cases. This is in keeping with previous studies [2,4,9]. Of the 75.7% of cases with physical lead points, 24 (64.9%) were tumours, still a characteristic finding in other clinical studies [1,2,4]. Furthermore, 54.2% (CI 35.1 – 72.1) of the tumours were malignant, representing 35.1% (CI 21.8 – 51.3) of all lesions (Table 4). This is comparable with the description of nearly half of intussusceptions in patients > 15 years being due to malignancy, documented by Barussaud et al. [23]. Other authors have also reported comparable contributions by malignant lesions to the overall intussusception burden. These include prevalences of 36% [4], 45% [9] and 46.6% [2]. This malignant property has a direct bearing in decision making during surgery. The 54.2% (CI 35.1 – 72.1) presence of malignancy, per se, among all tumours, is also comparable to some previously documented studies. These documented prevalences include 72.7% [4], 75% [2], 50% [9] and 22.2% [5].

Ten intussusception cases were caused by malignancies either involving the ileocolic area or the colon only (Table 3). Furthermore, 3 out of 15 cases (20%; CI 6.3 – 46.0) of ileocaecal/ileocolic intussusception had a malignancy. Wang et al. [19] reported relatively similar findings; 5/12 (42%) patients had malignant lesions in this type of intussusception. The commonest malignancy in our study was adenocarcinoma with 9/13 (69.3%; CI 42.0 – 87.7) of all malignancies. All of these occurred in the ileocolic or colocolic location. Barussaud et al. described 85% of purely colonic lesions as being adenocarcinomas [23]. Our findings compare well with this study and other studies, emphasizing the fact that the vast majority are adenocarcinomas. Another important issue regarding colonic intussusception was noted in a review study by Marinis et al. and other authors. They reported 66% of intussusceptions occurring in the colon as being secondary to malignancy [1,6,17]. As regards benign tumours, adenomas and lipomas (the most frequent forms [1,2,4]), were present in 6 and 4 cases respectively. All these were present in the ileocolic and colocolic types. They constituted 27% (CI 15.2 – 43.1) of all cases. Other comparable studies have reported prevalences of 18.2% [1] and 25% [9].

The presence of necrosis and/or gangrene in 27.0% (CI 15.2 – 43.1) of cases highlights the low association of sub-acute symptoms with these conditions. On the one hand, a more acute presentation would possibly show a higher frequency of necrosis/gangrene. On the other hand, a more chronic symptom presentation would tend to give a lower frequency, as described by Wang N et al. [9]. They described a lower frequency of 6.8%. Generally, few studies report specifically on this entity.

Because of the variability in clinical presentation and the varied nature of diagnostic imaging, it is common for the diagnosis to be made only at the time of laparotomy [6]. In this study we did not report on the pre-operative imaging investigations due to the non-uniform way in which they had been done. In most cases, no imaging was done. Otherwise, plain abdominal radiography and ultrasonography were the modalities used. No patient had an abdominal computerized tomography (CT) scan done. This very sensitive and specific investigation is very good for early diagnosis and decision making.

Most authors agree that laparotomy is mandatory in the treatment of AI, given that most cases have underlying pathological lesions. However, whether or not the intussusception should be reduced before resection remains controversial. Objections to reductions are theoretically based on: the possibility of intraluminal seeding and venous dissemination of malignant cells; possible perforation during manipulation; and the increased risk of anastomotic complications in the presence of oedematous and inflamed bowel [4]. The prevalence of 32.4% (CI 19.6 – 48.7) segmental bowel resections done among our patients, with and without reduction, is comparable with previous studies which described prevalences of 45% [5] and 43.9% [19]. There was a tendency to reduce enteric intussusceptions, partially or completely (Table 3). This is ostensibly because most surgeons go by the general principle of most enteric tumours being benign. No reduction was done in 14 cases (37.8%; CI 24.0 – 53.9), 11 of these involving the colon (colocolic or ileocolic). The colon-associated cases instead underwent definitive hemicolectomies. This observation, which also describes those who underwent resection without a prior attempt at reduction, is comparable to a 25% prevalence in a previous study [5].

It is suggested by many authors that if the underlying etiology and/or the lead point is suspected to be malignant, or if the resected area required without reduction is not massive, an en bloc resection of the intussusception should be considered. We observe that in patients with ileocolic, ileocaecal and colocolic intussusceptions, formal resections using appropriate oncologic principles were used in our centre. Primary anastomosis between healthy and viable tissue was done as is recommended [6,8,17,24]. Generally, large bowel should be resected without reduction because pathology is mostly malignant. For right-sided colonic intussusceptions, resection and primary anastomosis can be carried out even in unprepared bowel. However, for left-sided or rectosigmoid cases, resection with construction of a colostomy or performance of a Hartmann’s procedure, followed later by secondary anastomosis, is recommended especially in emergencies. We had one Hartman’s procedure performed.

Chronic intussusception, also present in this series, does not often allow for successful manual reduction to be performed due to thickening, fibrosis and cross-scarring within the intussusceptum [25]. Enteric intussusceptions due to benign lesions require only reduction and limited resection [19]. We encountered this in 4 cases of enteric intussusceptions, and one colocolic case. Reduction alone is adequate for idiopathic forms provided the bowel appears non-ischemic and viable [17]. Six of our patients had this done. However, one of them also had an incisional biopsy for a polyp (adenoma). Some patients at risk of short bowel syndrome require special consideration. We encountered a typical scenario in one case – intussusception (ileocolorectal, with anal protrusion) involving almost the entire colon and a sizeable length of ileum. A substantial length of gut was “milked” up to the limit of its reducibility, followed by resection [25].

There was no postoperative mortality. This may be explained by 3 factors: the small number of patients, the short post-operative observation duration (maximum 14 days), and the sub-acute/chronic nature of AI (generally not associated with short term, high mortality rates). Other authors too have reported no mortality [9,4]. Only one patient with ileocaecal adenocarcinoma, who underwent a right hemicolectomy, developed an enterocutaneous fistula. He was treated conservatively, and recovered 3 weeks postoperative.

Our study had its limitations. Firstly, it was a retrospective study, dependent on the accuracy of the patient case notes. Inadequate record keeping cost us 25 potential participants for analysis. Secondly, even where records were good, we could not get to the depth of the symptom of pain; continuous or ‘crampy’. Thirdly, the very important aspect of radiological investigation, vital in patient management, was not looked into. On top of this, the follow up period for the patients was not enough to fully assess their long term recovery. The exact duration of postoperative hospital stay was not included.

## Conclusion

AI is a rare but challenging condition for the surgeon. In our setting this condition seems not be as rare as in other settings. We have seen that 62 patients had intussusception during this 10 year period, though we could analyze only 37 because of unsatisfactory records. This is an average of over 6 cases per year. Preoperative diagnosis is usually missed or delayed because of nonspecific and often sub-acute symptoms, without the ‘pathognomonic’ (classic triad) clinical picture associated with intussusception in children. In our study we found it more practicable to look at intussusceptions diagnosed at operation, and then retrospectively analyze them.

Radiological imaging was not described due to non-uniformity of records. Abdominal CT is considered as the most sensitive imaging modality in the diagnosis of intussusception and distinguishes the presence or absence of a lead point [1,4,5]. Due to the fact that AI is often associated with malignant organic lesions, surgical intervention is necessary, usually requiring formal resection of the involved bowel segment. Reduction can be attempted in small bowel intussusceptions provided that the segment involved is viable, or a malignancy is not suspected. Surgeons should be familiar with the various treatment options because the real cause of the intussusception is often only accurately diagnosed at laparotomy, and a number of these cases have uniqueness about them [18,25].

## Competing interests

The authors declare that they have no competing interests.

## Authors’ contributions

PAO conceptualized the theme, collected the data and wrote the manuscript. CKO analyzed the data and co-wrote the manuscript. SCK edited the manuscript. All authors read and approved the final manuscript.

## Pre-publication history

The pre-publication history for this paper can be accessed here:

http://www.biomedcentral.com/1471-230X/14/86/prepub
